# Performance evaluation of AI-based caries detection technology and its educational training module: a dual-phase investigation

**DOI:** 10.3389/fdmed.2025.1741855

**Published:** 2026-01-29

**Authors:** Jennie Caldwell, Krunal Parekh, Brandon Crowther, Chiraag Gohel, Roberta Pileggi, A. Isabel Garcia, Mina Ghorbanifarajzadeh, Teresa A. Dolan, Anita Gohel

**Affiliations:** 1 Department of Oral and Maxillofacial Diagnostic Sciences, University of Florida College of Dentistry, Gainesville, FL, United States; 2 Department of Biostatistics and Bioinformatics, Computational Biology Institute, The George Washington University, Washington, DC, United States; 3 Overjet, Claymont, DE, United States; 4 The University of Jordan School of Dentistry, Amman, Jordan

**Keywords:** AI education, artificial intelligence (AI), caries, dental education, diagnosis, oral radiology

## Abstract

**Purpose:**

This objective of the study was to assess the accuracy of an AI-based caries detection system, Overjet Caries Assist, or OCA, (Overjet Inc. Claymont, DE, USA) and to analyze the efficacy of the AI-based caries detection training module in teaching dental students radiographic diagnosis of dental caries.

**Methods:**

Two independent calibrated observers evaluated 1604 proximal surfaces of teeth on intraoral bitewing radiographs and compared the findings to the OCA caries detection module. The sensitivity, specificity, positive and negative predictive values and diagnostic accuracy of the AI system were calculated. For the second part of the study, 82 first- and third-year dental students interpreted 10 intraoral bitewings for caries diagnosis before and after undergoing training with the OCA AI-caries detection training module. Non-parametric Wilcoxon signed-rank test was used to assess the difference between the students’ learning before and after the Overjet module training.

**Results:**

The average sensitivity for enamel lesions was found to be 0.69, while the average sensitivity for dentinal lesions was found to be 0.91. The average specificity for enamel lesions was found to be 0.99, while the average specificity for dentinal lesions was found to be 0.98. There was a 43%–46% increase in students’ ability to detect radiographic caries following the completion of the Overjet training module.

**Conclusion:**

The OCA module can be used as an effective tool for assisting diagnosis of caries among dental students. The Overjet training module is effective in training dental students’ radiographic diagnosis of caries.

## Introduction

Dental caries is a biofim-mediated prevalent chronic multifactorial infectious disease chiefly due to tooth-adherent cariogenic bacteria, mostly Streptococcus Mutans (1). Caries leads to progressive, localized tooth defects caused by the demineralization of dental hard tissues. This demineralization process is the result of a complex interaction of acid-producing bacteria, fermentable carbohydrates, saliva, and tooth (2). According to the NIH, nearly 90% of adults ages 20–64 have experienced tooth decay and one in four adults in this age range have untreated decay (3). Untreated decay will cavitate, leading to sensitivity, pain, and eventual pulpal infection (4). Proper identification, radiographic diagnosis, and treatment of caries constitute a fundamental aspect of the prevention, management and treatment of tooth decay.

The current treatment guidelines for dental caries favor early preventative intervention (5), and bitewing radiographic imaging is the standard of care of interproximal caries detection, especially for early signs of disease (6). However, teaching the reliable detection of radiographic carious lesions, especially in identifying the earliest signs of the disease, poses a considerable challenge (7). According to a systematic review of 117 studies, practitioners averaged a sensitivity of 0.24 for detecting any radiographic proximal carious lesions, including early enamel caries (incipient lesions) (8). Traditional methods of teaching radiographic caries in academic dentistry range from didactic lectures to readings and case presentations. Problem- based learning e-learning and other active teaching methodologies have provided little to no success in increasing the effectiveness of caries diagnosis in dental students (9).

Artificial Intelligence (AI) and Augmented Intelligence (AuI) are computer science tools increasingly being used to support clinical and administrative tasks that help in the delivery of dental care (10). AI is a discipline that deals with understanding and creating computer algorithms that are able to mimic human intelligence. AI has long been a part of medical imaging but has recently made its way into the dental imaging field (4).

AuI enhances human intelligence rather than replacing it. It plays a similar role as AI except it uses human intelligence elements in its procedure (10). AI models have been used in radiographic diagnosis of caries, vertical root fractures, apical pathology, lesions in the maxillofacial region and have been found to outmatch dentists in both performance and accuracy (11).

The use of AI in diagnostic medical imaging has shown accuracy in the identification of imaging abnormalities and could enhance disease detection and characterization (12). AI algorithms, particularly deep learning neural networks, have demonstrated significant fidelity in image-recognition tasks (13). These methods can be programmed for use in dental imaging specifically the identification of dental caries in intraoral images (14). Thus, the rapidly advancing field of AI presents promising interactive and self-learning solutions to address these clinical and educational challenges (15). Determining the true effectiveness of newly developed AI tools is essential to support evidence-based practice, underscoring the need for ongoing evaluation of these systems (16). Although such technologies may offer perceived diagnostic benefits, clinicians must remain aware of their inherent limitations. Independent assessments of key performance metrics—such as sensitivity, specificity, and predictive values—are crucial for understanding the reliability and appropriate clinical application of these AI-driven tools (17). Recent advancements in artificial intelligence (AI) and deep learning have significantly improved the performance of digital caries detection systems. Several recent studies have reported strong diagnostic accuracy of AI-based caries detection when applied to bitewing radiographs. Szabó et al. (2024) demonstrated that deep learning models achieved significantly higher sensitivity for proximal caries detection compared with general dentists, particularly for early enamel lesions (12). Valenzuela et al. (2024) reported that convolutional neural networks outperformed dental students in detecting non-cavitated proximal lesions, highlighting the potential of AI as a diagnostic adjunct (16). Similarly, Zhang et al. (2024) showed that transformer-based networks improved detection robustness across varying image quality conditions (17).

Applications of AI in dentistry are poised to change both clinical practice and the teaching methodology for dental students and residents. There is growing consensus that dental education should now include an understanding AI (18). It is vital that dentists become familiar with and appreciate the extent to which AI applications in dentistry can enhance patient treatment outcomes and communication (19). Embedding AI in dental education can augment the training process and enhance student engagement in learning. As AI has continued to expand and grow, AI-driven caries identification programs are making their way onto the market for use in clinical and educational environments. One of these AI tools currently available is the FDA-cleared Overjet Caries Assist (OCA) module (20). Overjet OCA utilizes various machine and deep learning techniques to offer a program designed for caries identification in bitewing radiographs, categorizing them as preventative or restorative. Overjet also offers an AI training module specifically designed to assist dental students in diagnosing caries (20). While the diagnostic performance of AI systems for caries detection has been increasingly documented, there remains a critical gap in the literature regarding the structured educational impact of AI-based training modules on dental student learning outcomes. Most published studies focus on algorithmic performance rather than on how AI-integrated platforms influence student diagnostic development, calibration, and confidence. Moreover, limited data exists comparing the effectiveness of traditional radiographic teaching with structured AI-assisted training.

Therefore, the present study uniquely addresses this gap through a dual-phase investigation evaluating both the diagnostic accuracy of the Overjet Caries Assist (OCA) system and the educational impact of its AI-based training module on first- and third-year dental students. In this study we sought to assess two aspects of such AI tools: the sensitivity and specificity of caries detection for the OCA program and the effectiveness of the Overjet training module in enhancing student learning of radiographic caries diagnosis. We propose the integration of such tools will improve models of augmented radiology education.

## Materials and methods

The study was performed in two phases. First, an assessment of the sensitivity and specificity of the Overjet Caries Assist (OCA) program was performed. Second, an evaluation of the Overjet training module and its effectiveness as a caries teaching modality was evaluated.

### Assessment of sensitivity and specificity of overjet caries assist

In this phase of the study, the sensitivity and specificity of the OCA program was assessed. One oral and maxillofacial radiology resident and a fourth year dental student were calibrated by a board-certified oral and maxillofacial radiologist with more than 25 years of experience through the review and discussion of five radiographic cases to confirm agreement on the presence of interproximal decay.

**Figure 1:**
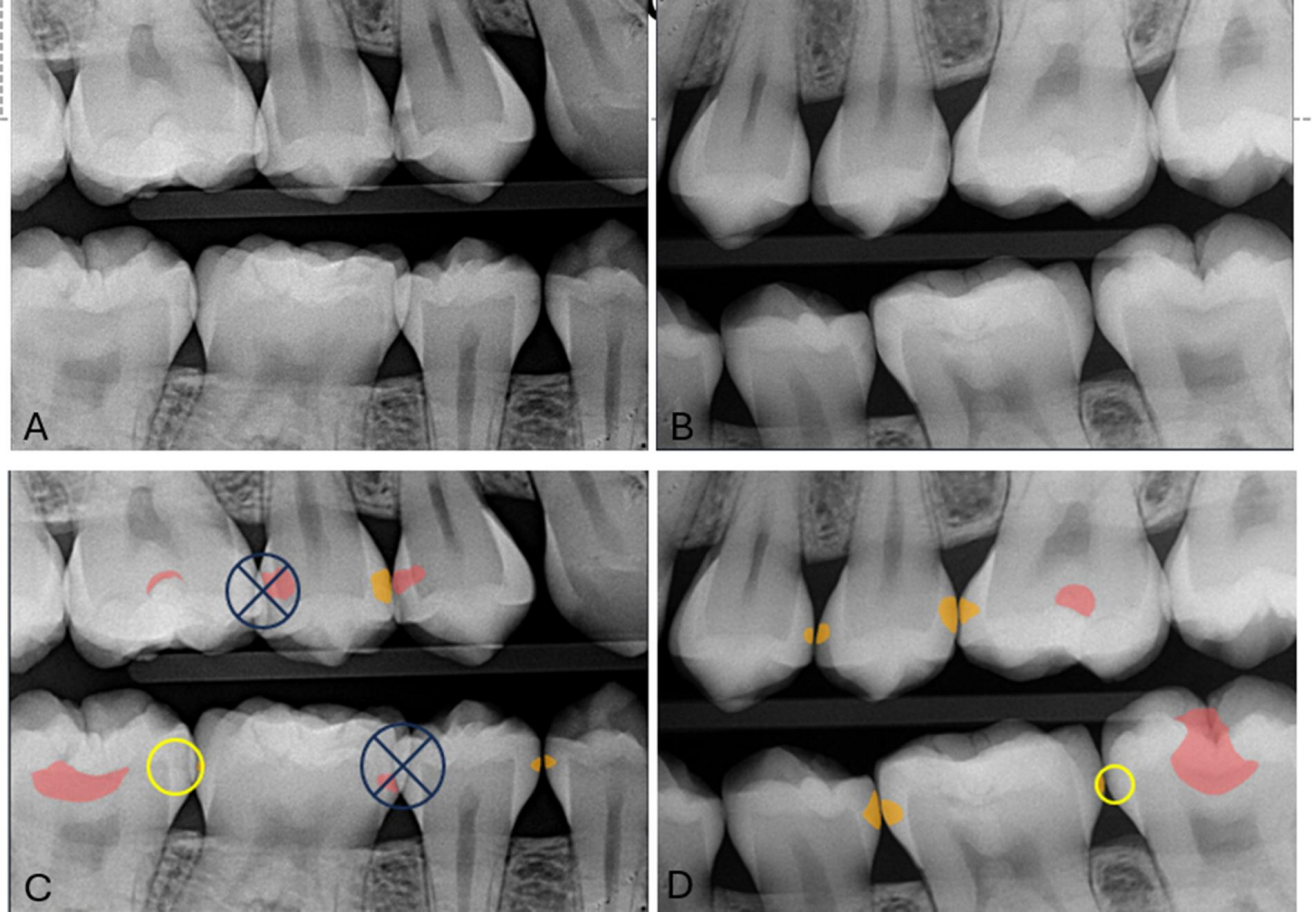
Bitewing radiographs **(A,B)** without OCA interpretation. Images **(C,D)** show OCA program applied. “early enamel caries” caries labeled in orange and “treatable” caries labeled in red. The yellow circle indicates caries false negative and the X indicates surfaces with overlap that did not qualify for consideration.

A protocol was determined for accessing bitewings, applying the AI program, and recording the data. No more than ten cases were to be read a day to prevent eye fatigue. The Dell U3219Q 3840 × 2,160 resolution 8-bit monitor was used. Each examiner assessed 100 bitewing images (for 200 total assessed images, 1,142 surfaces). They first evaluated for early enamel caries, then applied the OCA program. For each image, they documented the total teeth, total surfaces, and caries correctly identified, incorrectly identified, and missed by the program. From this recorded data, sensitivity and specificity were calculated for each image then averaged for each examiner. The inclusion criteria for the number of teeth in an image was: at least 50% of one proximal surface is visualized and at least one visualized surface is non-restored. The inclusion criteria for a surface to be evaluated was interproximal surface, non-restored, at least 50% captured in image, and less than 50% overlap (see Figure 1). The process was then repeated with each examiner assessing 100 images for dentinal caries, totaling 200 images. To determine inter-operator reliability, each examiner individually assessed thirty of the same cases. To confirm intra-operator reliability, the same thirty cases were again assessed thirty days later.

### Effectiveness of overjet training module on student learning

In this phase of the study, 10 intra-oral bitewing radiographs with 36 interproximal carious lesions and four occlusal carious lesions were selected for evaluation by the students. The inclusion of 40 confirmed lesions per assessment ensured sufficient event frequency for stable sensitivity estimation and minimized sparse-data bias. The gold-standard for this study was established by a consensus of Oral and Maxillofacial Radiology (OMR) faculty at the with more than twenty-five years of experience and a calibrated OMR intern. Each operator evaluated images separately, and the consensus was obtained by discussion for any disagreement. Agreement between the two established by independent grading prior to any discussion was assessed by Cohen's *κ* coefficient.

A total of eighty-two dental students, twenty D1 and sixty-two D3 students, volunteered to participate in the study. Students were invited by email and in-class announcements. Participation was voluntary and had no impact on course grading. The inclusion criteria consisted of active enrollment as a D1 or D3 student and completion of the foundational radiology didactic curriculum appropriate to their year level. Exclusion criteria included prior exposure to the Overjet Caries Assist (OCA) software, previous clinical use of AI-based caries detection tools, and incomplete participation in both the pre- and post-training assessments. A lesion-based power analysis was performed for paired sensitivity outcomes using a two-tailed α of 0.05% and 80% power. With 40 confirmed carious lesions evaluated per student in a paired pre–post design, detection of a 10% absolute improvement in sensitivity required a minimum of 34 students. Detection of a 20% improvement required 16 students. The final sample of 82 students substantially exceeded these thresholds, providing robust statistical power to detect even modest changes in diagnostic performance while accounting for within-reader clustering.

The students initially charted the radiographic caries independently. All students then completed a standardized training module on the Overjet Caries Assist (OCA) AI platform before the post-training assessment. Each training session lasted 1 h and included small groups of 5–6 students. Students first independently annotated carious lesions on 10 standardized bitewing radiographs as a baseline (pre-training) assessment. This was followed by a structured PowerPoint presentation introducing artificial intelligence and its application to radiographic caries detection, including an overview of the Overjet platform interface and functionality. A series of calibrated 10 real-time full-mouth-series (FMX) sample cases with known caries status was used to standardize diagnostic thresholds, followed by independent practice in which students identified carious lesions with and without AI assistance and compared their interpretations to reference standards. Throughout the module, faculty and residents reinforced the need for critical evaluation of AI findings and confirmation using conventional radiographic criteria. After completion of the training session, students re-annotated the same initial 10 bitewing radiographs previously evaluated, enabling direct comparison of diagnostic performance before and after OCA training. The diagnostic process was conducted using the Dell Ultrasharp 1,920 × 1,080 resolution U2422H 23.8” monitors in a radiology reading room with ambient background lighting.

The true positives (TP), false positives (FP), true negatives (TN), and false negatives (FN) were determined by comparing them to the radiologist's diagnosis, which served as the reference standard. Sensitivity (SE), specificity (SP), positive predictive value (PPV), and negative predictive value (NPV) were calculated for both enamel and dental caries. Accuracy (ACC) was defined as ACC = (TN + TP)/(TN + TP + FN + FP). F1 Scores were calculated as F1 = 2TP/(2TP + FP + FN). The non-parametric Wilcoxon signed-rank test was used to assess the difference between the students’ learning before and after the Overjet module training. Statistical significance was set at *p* < 0.05. Intraclass correlation coefficient with 95% confidence intervals was calculated.

## Results

### Assessment of sensitivity and specificity of overjet caries assist

Intrarater reliability for the two raters for measurements performed was greater than 0.9.

**Figure 2:**
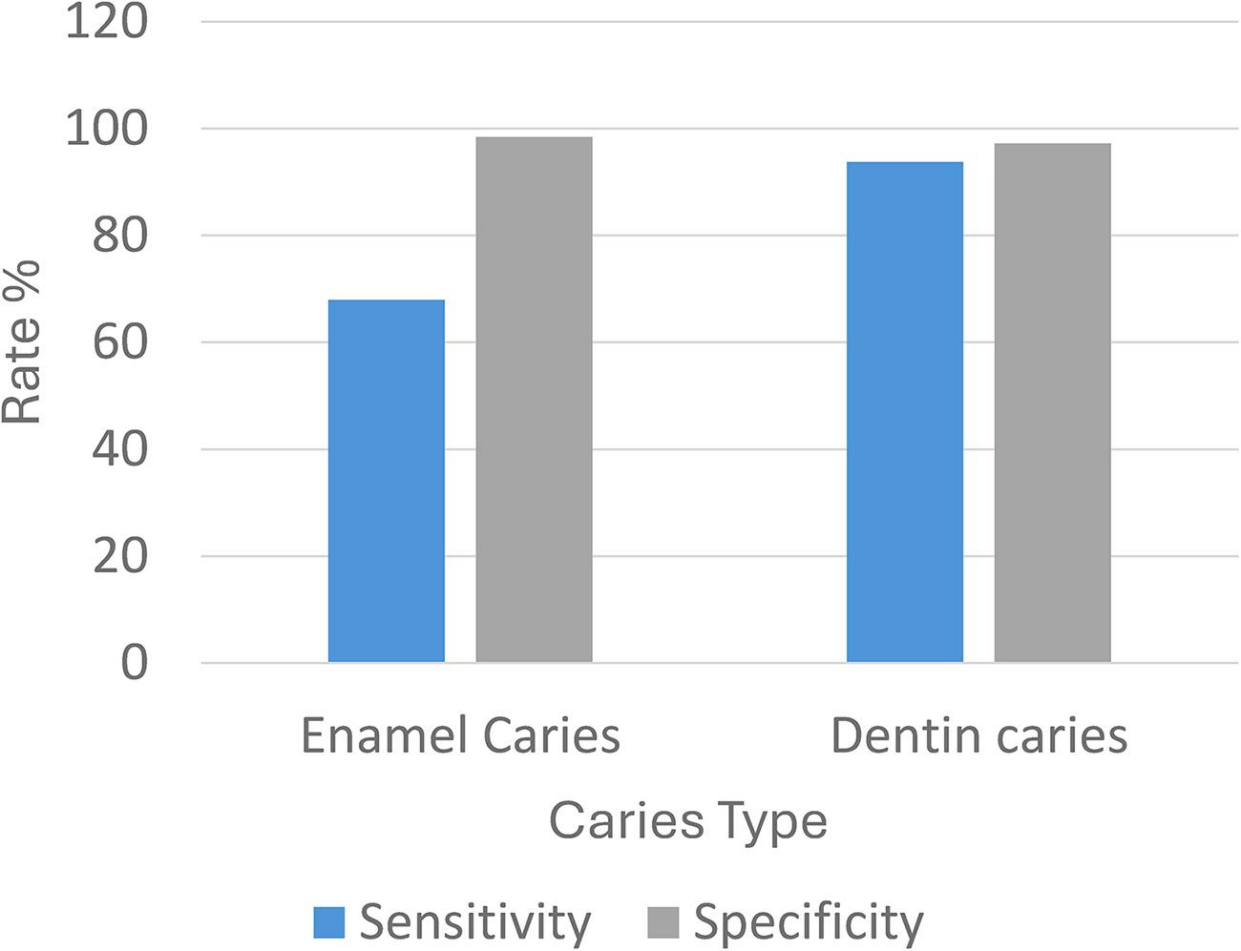
Pre-training and post-training sensitivity and specificity for interproximal enamel and dentinal caries detection using the overjet caries assist (OCA) module. The figure demonstrates high diagnostic performance of the OCA system, with significantly greater sensitivity for dentinal lesions compared to enamel lesions.

The average sensitivity for detecting enamel lesions was 69% (95% CI: 63%–73%), while for dentinal lesions, it was significantly higher at 91% (95% CI: 94%–96%). The average specificity was 99% (95% CI: 98%–99%) for enamel lesions and 98% (95% CI: 97%–99%) for dentinal lesions (see Figure 2). The positive predictive value (PPV) was 94% for enamel lesions and 93% for dentinal lesions. The negative predictive value (NPV) was 85% for enamel caries and 96% for dentinal caries.

The accuracy for detecting enamel caries was 87%, with an F1 score of 0.78. For dentinal caries, the accuracy was higher at 96%, with an F1 score of 0.93. The false positive rate was 1.9% for both enamel and dentinal caries (see Table 1).

**Table 1:** Diagnostic performance metrics for OCA-assisted detection of interproximal enamel and dentinal caries, including true positives, true negatives, false positives, false negatives, accuracy (ACC), F1 score, positive predictive value (PPV), and negative predictive value (NPV).

Diagnostic accuracy of OCA-assisted caries detection								
Caries extent	True positive	True negative	False Positive	False Negative	ACC %	F1 score	PPV	NPV
Enamel caries	245	689	13	122	87.37	0.78	94	85
Dentin Caries	280	754	15	27	96.09	0.93	93	93

Diagnostic accuracy of OCA-assisted caries detection.

ACC, accuracy; F1, F1 score; SE, sensitivity; SP, specificity; NPV, negative predictive value; PPV, positive predictive value; CI, confidence interval.

### Effectiveness of overjet training module on student learning

The findings of this study reveal an increase in student learning outcomes related to radiographic caries diagnosis when utilizing the Overjet AI training module. Before training with the AI module, D1 students were able to identify about 25% of the caries which increased to 35% after the training. The D3 students were able to identify 53% of the caries before training and this increased to a statistically significant 75% after the training (*p* < 0.05).

**Figure 3:**
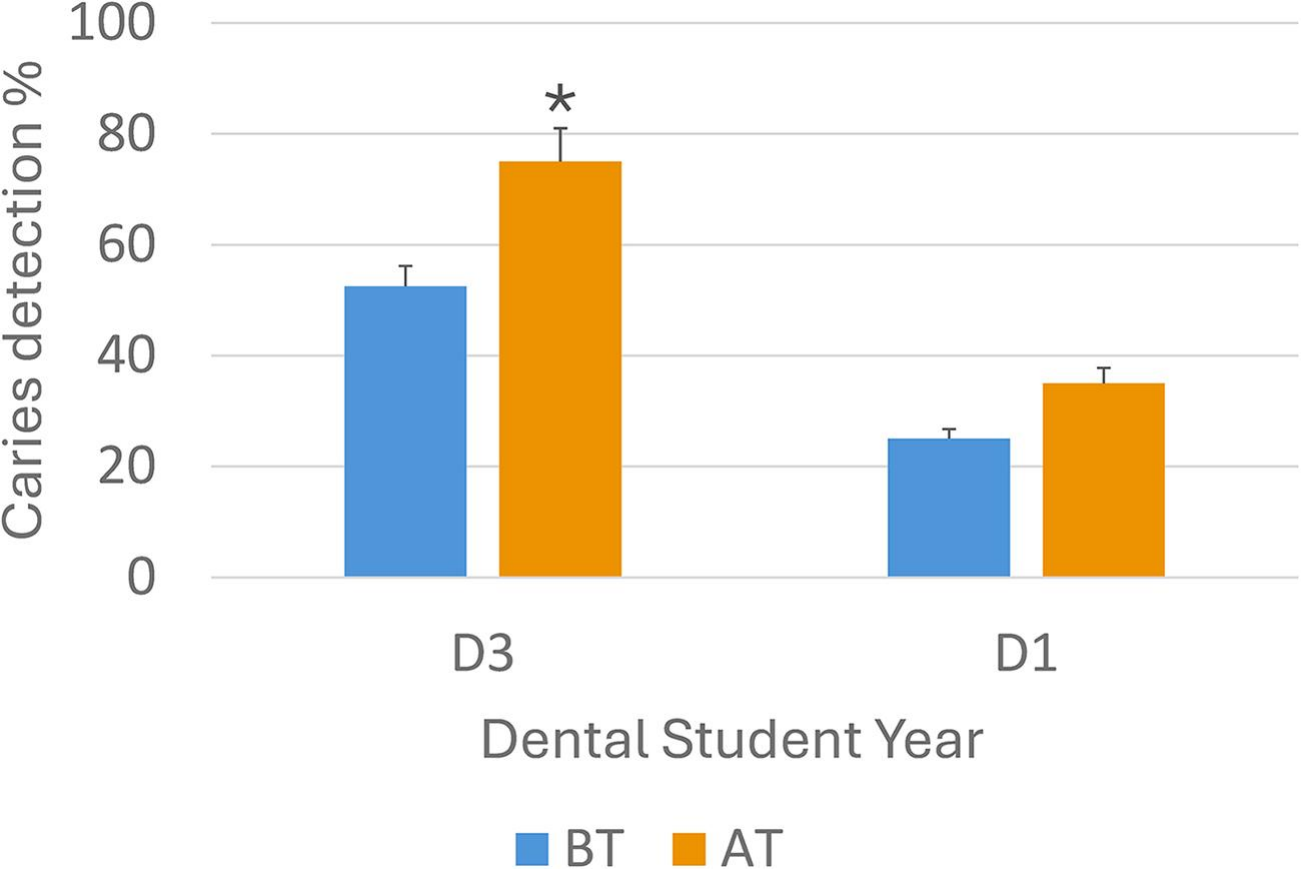
Chart showing the percentage of correctly identified caries before and after the AI module was used with D1 and D3 students respectively with respective standard deviation. BT, before training with the AI training module; AT, after training with the AI training module (*p* < 0.05).

Among the D1 students, there was a 43% improvement in the identification of carious lesions, accompanied by a 5% false-positive rate post-training. Similarly, D3 students experienced a significant 46% increase in their ability to identify carious lesions, with a 7% false-positive rate after completing the Overjet module training (see Figure 3). D3 students scored statistically better in both initial and post-training identification of proximal carious lesions compared with the D1 students.

## Discussion

Accurate diagnosis of carious lesions is a critical component of clinical dentistry, as it directly influences treatment outcomes in both vital and endodontically treated teeth. The extent and location of carious involvement not only affect the prognosis of non- endodontically treated teeth but also play a significant role of root canal treated teeth, where non-restorability due to extensive caries remains a leading cause of extraction (21). Interproximal caries, in particular, has been implicated in the pathogenesis of pulpal calcification, such as pulp canal obliteration (PCO), highlighting the importance of early detection and intervention (22). Recent histobacteriological evidence indicates that even after selective excavation to “leathery” or “firm” dentin, substantial bacterial infiltration persists within the dentinal tubules and pulpal tissues (23). This residual microbial presence elicits chronic subclinical pulpal inflammation, underscoring that the apparent arrest of caries does not equate to microbial quiescence. Non-cavitated interproximal white spot lesions also demonstrated bacterial biofilm in the dentin (24). Therefore, comprehensive detection and thorough removal of carious tissue are essential to prevent pulpal pathology and to support biologically sound, evidence-based clinical decision-making (23, 24).

In general, the sensitivity of detection of proximal carious lesions by humans ranges from 24 to 43% and specificity ranges from 89%–97%. Our results indicate that OCA is overall accurate, with higher sensitivity and specificity on proximal carious lesions and markedly high sensitivity for dentinal lesions. Thus, the OCA AI model performed reliably as a tool for assisting in the diagnosis of caries. Periodic repetition of the study would be advised to confirm the continued efficacy and reliability of the AI model and to also identify any data drift that may lead to the model performance degradation over time. It also may assist in understanding how the program learns and improves over time.

Artificial Intelligence (AI) is permeating every aspect of our lives, including the dental profession. AI can assist in detecting caries, periodontal bone loss, periapical lesions, and other pathologies on radiographs. An understanding of how these tools work will enable dentists to interpret and verify AI-generated findings responsibly. The collaboration between a dentist's expertise and AI systems (AuI), where AI has a supportive role in diagnostic decision making will lead to better diagnostic and treatment outcomes.

With AI-driven tools becoming more broadly adopted among practicing dentists, it falls on us as dental educators to ensure our students are properly familiarized with the programs and integrate them into the curriculum. Dentists are ethically and legally responsible for diagnosis and treatment. Overreliance on AI systems may lead to diagnostic deskilling of our students. We need to train our future clinicians on the ethical use of AI and also to avoid automation biases (25). Dental students need to be trained on the risks of underdiagnosis and overtreatment and avoid diagnostic errors, missed conditions, or inappropriate treatments. Dental school faculty need an understanding of AI fundamentals to integrate AI into their didactic and clinical teaching. They should also be equipped to critically evaluate the accuracy, reliability and the ethical considerations of different AI tools. An effective AI curriculum must include faculty development and calibration to ensure educators can prepare future dentists with the foundational knowledge and practical skills necessary for the responsible and successful integration of AI into clinical practice (26).

The integration of AI into dental education, exemplified by the Overjet Caries Assist training module, is transforming the training of our dental students in radiographic caries diagnosis. It is our experience that this AI tool is able to provide real-time feedback and student engagement, enabling precise self-learning for effective identification of carious lesions. Increasingly, the scientific literature, including studies by Sanjeev B Khanagar et al., 2021 underscores the positive impact of AI in dentistry, particularly in diagnostics (11).

At the time of this study, the participating D3 students were in the middle of their Spring semester. They already had completed approximately 9–10 months of clinical experience in addition to didactic courses on caries diagnosis during their first and second year. Meanwhile, the D1 students had only been exposed to caries diagnosis as part of didactic courses taught using traditional teaching methods. Traditional methods of teaching combined with clinical caries diagnostic experience has a greater impact, as the D3 students scored better in initial caries diagnosis compared to D1 students who had only received didactic training. Yet, the addition of AI training using OCA had a more pronounced impact on both D1 and D3 students. The observed improvement in post-training diagnostic performance suggests that AI-assisted educational platforms may enhance radiographic pattern recognition by reinforcing visual cues associated with early-stage carious lesions. diagnostic experience, highlighting the value of early AI exposure in preclinical training.

Our students expressed a desire for inclusion of AI-driven tools in the dental curriculum, aligning with current trends of AI adoption in dentistry. Our future plans are to expand the study to include more students, aiming to assess the sustained impact of the OCA integration over one year. It is hoped that our results contribute to the ongoing scientific and academic discussions to encourage greater recognition and assessment of the role of AI as an integral part of the dental learning experience.

Conventional teaching of radiographic caries interpretation in dental education is primarily delivered through didactic lectures, static image review, and limited clinical exposure during routine radiology practical sessions. While these methods establish foundational knowledge, they often lack individualized feedback, real-time error correction, and standardized calibration. In contrast, the OCA AI-based training module provides immediate visual feedback, automated lesion detection overlays, and structured self-directed learning, allowing students to iteratively compare their interpretations with validated AI outputs. This structured, interactive approach likely explains the significantly higher post-training diagnostic gains observed in both D1 and D3 cohorts.

These findings are consistent with prior investigations evaluating AI-assisted dental education. Valenzuela et al. and Szabó et al. similarly reported significant improvements in student and practitioner diagnostic accuracy following exposure to deep learning–based training systems (12, 16). Porumb et al. further demonstrated improved intra-observer consistency when AI feedback was incorporated into radiographic interpretation (27). However, unlike these studies, the present investigation uniquely combines validation of algorithmic diagnostic performance with formal educational outcome assessment within the same cohort.

Using the AI module will help educators train students to be competent in diagnosis of common dental diseases, and this in turn will make them better practitioners. Our vision is to leverage this innovative teaching method to enhance both student learning outcomes and the delivery of oral health care.

The incorporation of AI as an adjunct to conventional radiological diagnosis needs further assessment to evaluate its performance over time. Following their training, we observed a tendency by the dental students to overdiagnose caries with a 5%–7% false positive rate, or FPR. The FPR with the OCA system is approximately 1.9%. Hence, faculty expertise and supervision will be necessary and using AI as a complementary role (AuI) and not as a substitute for radiographic diagnosis to ensure the best possible patient outcome.

## Conclusion

The Overjet Caries Assist demonstrated strong utility in accurately identifying the presence and absence of interproximal caries on intraoral radiographs. Additionally, the Overjet Training Module produced a substantial improvement in students’ radiographic caries detection performance. Collectively, these AI-driven tools enhanced students’ diagnostic competence and clinical decision-making, which are essential for the appropriate and effective management of dental caries. These findings support the growing role of artificial intelligence as a valuable adjunct to traditional dental education and suggest that structured AI-based training may play an increasingly important role in preparing future clinicians for technology-integrated practice.

## Data Availability

The data analyzed in this study is subject to the following licenses/restrictions: Available by request. Requests to access these datasets should be directed to jcaldwell@dental.ufl.edu.
